# Is conservative management of ductal carcinoma in situ risky?

**DOI:** 10.1038/s41523-022-00420-2

**Published:** 2022-04-28

**Authors:** Lan Zheng, Yesim Gökmen-Polar, Sunil S. Badve

**Affiliations:** 1 Indiana University School of Medicine, Indianapolis, IN USA; 2 Department of Pathology and Laboratory Medicine, Emory University School of Medicine, 1364 Clifton Road, H 184, Atlanta, GA 30322 USA

**Keywords:** Breast cancer, Cancer prevention

## Abstract

Nonsurgical management of ductal carcinoma in situ is controversial and little is known about the long-term consequences of this approach. In this study, we aimed to determine the risk of (a) upstaging to invasive carcinoma at excision and (b) ipsilateral breast cancer events in patients who might have been eligible for nonsurgical management of DCIS trials. Data from women aged 20 years or older with a biopsy diagnosis of DCIS between January 1, 2010 to December 31, 2014 were collated. The women underwent biopsy and surgical resection (lumpectomy or mastectomy) and were treated with radiation or endocrine therapy as per treating physicians’ choice. The development of ipsilateral breast cancer events (IBEs) was analyzed in patients with at least 5 years of follow-up after standard of care therapy for DCIS. Subset-analysis was undertaken to identify the incidence of IBEs in patients eligible for nonsurgical management trials. The study population consisted of 378 patients with matched cases of biopsy and surgical excision. The overall upstaging rate to IBC was 14.3 and 12.9% for COMET, 8.8% for LORIS, and 10.7% for LORD trial “eligible” patients. At 5 years of follow-up, ~11.5% of overall and trial eligible patients developed IBEs of which approximately half were invasive IBEs. In conclusion, women with DCIS who would have been eligible for nonsurgical management trials have a significantly high risk of developing ipsilateral breast events within 5 years of diagnosis. Better selection criteria are needed to identify DCIS patients who are at very low risk for the development of IBC.

## Introduction

Ductal carcinoma in situ (DCIS) of the breast is a group of complex and heterogeneous lesions. Currently, the incidence rate of DCIS in a general population of women aged 40 years and older is about 31.5 cases per 100,000 person-years and represents ~20–25% of all new breast neoplastic lesions diagnosed^1^. There is marked heterogeneity in the clinical presentation, histologic features, genetic abnormalities, and biologic potential of DCIS. Clinically, DCIS can present as a palpable mass, nipple discharge, or Paget disease. The widespread use of population-based screening mammography has resulted in more than 85% of asymptomatic DCIS cases^2^. However, despite substantial increases in the number of cases of pre-invasive and early-stage breast cancer detected, screening mammography has only marginally reduced the incidence of advanced-stage cancer at diagnosis^3^. Moreover, DCIS is a precursor and many lesions will never progress to invasion in a patient’s lifetime^2,4^. As a result, there is strong interest in studying the factors which may reliably distinguish high-risk DCIS from indolent cases; the latter may potentially be managed by nonsurgical/active surveillance strategies.

The natural history of DCIS and factors associated with the development of local recurrence/ipsilateral breast cancer events (IBE) are poorly understood. DCIS, diagnosed on biopsy, has been associated with “upstaging” to invasive breast carcinoma (IBC) on excision in 8 to 42.7% of cases^5–12^. The risk factor associated with DCIS upstaging to IBC includes older age at diagnosis^8^, family history of breast cancer^8^, palpable mass^7^, large tumor size^13^, high-grade DCIS^14^, and presence of necrosis^15^. Analysis of the ECOG E5194 clinical trial showed that low-grade DCIS was associated with a low risk of development of IBE^16^. A similar analysis of the UK/ANZ DCIS trial identified a number of pathological features to be associated with local recurrence^17^. These included high cytonuclear grade, larger lesion size, growth pattern, presence of necrosis or chronic inflammation, incompleteness (or uncertainty of completeness) of excision, and smaller margin width. Data such as these lay the foundation for de-escalation therapies in DCIS. Currently, there are three prospective, randomized, phase III clinical trials seeking to answer the question of whether low risk DCIS can be safely treated with nonsurgical therapeutic regimens. The COMET (Comparison of Operative to Medical Endocrine Therapy for low-risk DCIS), LORIS (Low RISk DCIS), and LORD (Low Risk DCIS) studies ask the question of whether active surveillance in screen-detected low-grade DCIS diagnosed on biopsy can be safely managed by nonsurgical means.

De-escalation therapies for DCIS are controversial. Morrow and Winer argue that avoidance of surgery would mean more intensive imaging follow-up, a higher percentage of false positives, and more biopsies^18^. On the other hand, Hwang et al. argue that the risk and consequences of missing an invasive focus at excision are overestimated^19^. Although there are a number of studies that have analyzed the question of upstaging^5,10–13^, we were unable to identify any that had looked at the possible long-term impact of de-escalation therapies. We studied patients who could have been candidates for these de-escalation trials with the following goals (a) to determine the upstaging rates of invasive breast carcinoma (IBC) and (b) to identify the 5-year risk of IBC.

## Results

### Clinical and histological characteristics of DCIS at core biopsy

The clinical and histological characteristics of the 378 DCIS cases diagnosed on breast biopsy during the period 2010 to 2014 are shown in Table 1. Forty-four of patients were <45 years and 335 patients were ≥45 years. About 72% of patients are Caucasian and 18% are African-American. Slightly more than one-third (38%) of patients had a family history of breast cancer. Clinically, about 14.8% of patients had a mass lesion on imaging and 21.5% of patients had more than one lesion. On histological exam of the core needle biopsies, 9% of DCIS lesions were graded as low-grade, 49.7% as intermediate-grade, and 41.3% were graded as high-grade. Focal necrosis or comedo necrosis was identified in 70.6% of biopsied lesions. Expression of ER was noted in 82% and PR in 71.7% of cases. Of the 378 patients who underwent surgical excision 194 (51.3%) had breast-conserving surgery while 184 (48.7%) had a mastectomy. After surgery, 17.7% of patients received radiation therapy only, 17.2% received hormonal therapy only, and 31.5% of patients received both radiation and hormonal therapies.

**Table 1:** Clinical and pathological characteristics of patients diagnosed with DCIS on biopsy and parameters associated with upstaging to invasive cancer at surgery.

	Entire population (*n* = 378)	No upstage (*n* = 324)	Upstage (*n* = 54)	*P value*	OR (95% CI)
Age (years, mean [range])		58.6 (29–90)			
<45 years	44 (11.6%)	36 (11.1%)	7 (13%)	0.50	1.42 (0.51–3.93)
≥45 years	335 (88.6%)	288 (88.9%)	47 (87%)		
Race					
Caucasian	272 (72%)	230 (71%)	42 (77.8%)	0.40	1.49 (0.59–3.78)
African-American	68 (18%)	59 (18.2%)	9 (16.67%)		
Other	38 (10%)	35 (10.8%)	3 (5.6%)		
History of breast cancer					
Yes	144 (38%)	54 (16.6%)	21 (38.8%)	0.54	1.24 (0.62–2.50)
No	234 (62%)	270 (83.3%)	33 (61.1%)		
Clinical presentations					
Mass	56 (14.8%)	44 (13.6%)	12 (22.2%)	**0.01**	3.20 (1.42–7.18)
Asymptomatic %	315 (83.3%)	280 (86.4%)	42 (77.8%)		
DCIS grade					
Low	34 (9%)	30 (9.3%)	4 (7.4%)	0.45	0.59 (0.14–2.35)
Intermediate	188 (49.7%)	162 (50.0%)	26 (48.1%)		
High	156 (41.3%)	130 (40.1%)	23 (44.5%)	0.07	2.02 (0.94–4.32)
At least focal Necrosis					
Yes	267 (70.6%)	241 (74.4%)	35 (64.8%)	0.19	0.57 (0.24–1.32)
No	94 (29.4%)	80 (24.7%)	19 (35.2%)		
Number of lesions					
Single lesion	301	262 (80.9%)	39 (72.2%)		
Multiple lesions	77	62 (19.1%)	15 (27.8%)	0.07	2.02 (0.94–4.32)
Estrogen receptor (ER)					
Positive	311 (82%)	266 (82.1%)	46 (85.2%)		
Negative	64 (16.9%)	55 (17%)	8 (14.8%)	0.27	5.73 (0.39–3.26)
Progesterone receptor (PR)					
Positive	271 (71.7%)	231 (71.3%)	40 (74.1%)		
Negative	104 (27.3%)	90 (27.8%)	14 (25.9%)	0.82	1.13 (0.39–3.26)

Surgical excision, lumpectomy, or mastectomy, upstaged 16.6% of total cases of invasive carcinoma (Table 1). Twelve of 54 patients had presented with a mass lesion on imaging. Histologically, these cases had been graded as a low grade in 7.4% (4/54); an intermediate-grade in 48.1% (26/54), and a high grade in 44.5% (24/54) on core needle biopsy. At least focal necrosis was present in 64.8% (35/54) biopsies in these cases.

Table 1 also shows the multivariate associations between upstaging at surgery and clinical and histological characteristics in 378 cases. An imaging identified/ palpable mass lesion was associated with an increased risk of upstaging (OR = 3.196, 95%CI: 1.42–7.18, *p* = 0.01). Moreover, patients who underwent mastectomy tended to show an increased upstaging risk of invasive carcinoma (OR = 1.99, 95%CI: 0.99–3.96, *p* = 0.05). Similarly, patients with multiple lesions and histologic high-grade DCIS had a strong trend of upstaging to invasive carcinoma (*p* = 0.07). All remaining characteristics were not significantly associated with upstaging to invasive carcinoma.

### Ipsilateral breast cancer events (IBE) at 5 years of follow-up

Follow-up data with 5 years was available on 243 patients, who were not upstaged to invasive carcinoma after surgical excision. 27 (11.1%) patients developed another ipsilateral breast event within 5 years of follow-up (Table 2). Among them, 59.3% (16/27) developed DCIS, 37% (10/27) developed invasive carcinoma, and 3.7% (1/27) had LCIS. Table 2 shows a multivariable analysis of clinical and histological characteristics associated with second breast event in 243 non-upstaged cases at 5 years follow-up and in cases. None of these characteristics were significantly associated with IBEs in multivariate analysis.

**Table 2:** Clinical and pathological characteristics of patients with DCIS who developed Ipsilateral breast events after at least 5 years follow-up.

	Event free (*n* = 216)	Ipsilateral breast events (*n* = 27)	*p* value	OR (95% CI)
Breast surgery				
Lumpectomy	118 (54.6%)	17 (63.0%)		
Mastectomy	137 (63.4%)	10 (37.0%)	0.10	0.40 (0.13–1.21)
Age				
<45 years	19 (8.8%)	4 (14.8%)	0.15	2.97 (0.67–13.01)
≥45 years	197 (91.2%)	23 (85.2%)		
Race				
Caucasian	212 (98.1%)	18 (66.7%)	0.69	1.28 (0.38–4.30)
African-American	153 (70.8%)	6 (22.2%)		
Family history of				
Breast cancer	76 (35.2%)	9 (33.3%)	0.52	0.73 (0.27–1.94)
Negative	132 (61.1%)	16 (59.3%)		
Clinical presentations				
Mass	32 (14.8%)	5 (18.5%)	0.23	0.38 (0.07–1.88)
No mass lesion	184 (85.2%)	22 (81.5%)		
Number of lesions				
Single lesion	178 (82.4%)	21 (77.8%)		
Multiple lesions	39 (18.1%)	6 (22.2%)	0.59	1.40 (0.41–4.81)
Necrosis				
Yes	155 (71.8%)	21 (77.8%)	0.84	0.51 (0.16–1.61)
No	59 (27.3%)	6 (22.2%)		
DCIS grade				
low	19 (8.8%)	2 (7.4%)	0.94	0.94 (0.17–5.24)
intermediate	116 (53.7%)	14 (51.9%)		
high	80 (37.0%)	11 (40.7%)	0.85	1.11 (0.37–3.31)
Estrogen receptor (ER)				
Positive	188 (87.0%)	21 (77.8%)	0.90	1.39 (0.01–23.09)
Negative	28 (13.0%)	6 (22.2%)		
Progesterone receptor (PR)				
Positive	210 (97.2%)	17 (63.0%)	0.11	0.36 (0.10–1.26)
Negative	49 (22.7%)	10 (37.0%)		
Radiation therapy				
Yes	114 (52.8%)	15 (55.6%)	0.19	2.03 (0.71–5.84)
No	97 (44.9%)	12 (44.4%)		
Hormonal therapy				
Yes	115 (53.2%)	15 (55.6%)	0.73	1.2 (0.42–3.46)
No	96 (44.4%)	12 (44.4%)		

### Incidence of upstage to invasive carcinoma in trial eligible patients

According to the eligibility criteria^20–22^, there were 163 patients eligible for the COMET Trial, 34 patients for the LORIS Trial, and 56 patients for the LORD Trial. The clinicopathologic characteristics of the entire cohort and the trial eligible patients is shown in Table 3. As is evident from the table, the trial eligible subsets were comparable to the patient population in racial distribution and had similar rates of mastectomy. Table 3 also shows the frequency of the radiation and endocrine therapy in the overall population and the patients with IBE. Of note, there was no significant differences in the use of radiation or endocrine therapy (*p* = 0.19 and 0.73 respectively).

**Table 3:** Clinicopathological and treatment characteristics of patients in a subset of patients who would have met eligibility conditions for clinical trials using nonsurgical approaches.

	COMET eligible (*n* = 163) No. (%)	LORIS eligible (*n* = 34) No. (%)	LORD (new) eligible (*n* = 56) No. (%)
Age (years, mean [range])	58 [40–86]	60 [46–81]	61.4 [45–86]
<45 years	0	0	0
≥45 years	163	34	56
Race			
Caucasian	120 (73.6%)	16 (47.0%)	39 (69.6%)
African-American	26 (16%)	5 (14.7%)	9 (16%)
Other/missing	17 (10.4%)	13 (38.2%)	8 (14.3%)
Family history of			
Breast cancer			
Yes	59 (36.2%)	0	19 (33.9%)
No	104 (63.8%)	34 (100%)	37 (66%)
Clinical presentations			
Mass	0	0	0
Nipple Discharge	0	0	0
Asymptomatic %	163(100%)	34(100%)	56(100%)
DCIS grade			
low	25 (15.3%)	11 (32.4%)	19 (33.9%)
intermediate	138 (84.9%)	23 (67.7%)	37 (66%)
high	0	0	0
At least focal Necrosis	102 (62.6%)	0	0
Breast surgery			
Lumpectomy	87 (53.4%)	16 (48.2%)	28 (50%)
Mastectomy	76 (46.6%)	18 (52.9%)	28 (50%)
Estrogen receptor (ER)			
Positive	163 (100%)	32 (94.1%)	53 (94.6%)
Negative	0	2 (5.9%)	3 (5.4%)
Progesterone receptor (PR)			
Positive	161 (88.8%)	30 (87.9%)	48 (85.7%)
Negative	2 (1.2%)	4 (12.1%)	8 (14.3%)
Adjuvant therapy			
Radiation therapy only	29 (17.8%)	5 (14.7%)	9 (16.1%)
Hormonal therapy only	33 (20.3%)	3 (8.8%)	9 (16.1%)
Both	57 (35.0%)	15 (44.1%)	22 (39.3%)
Upstaging to invasion			
Invasive carcinoma	21(12.9%)	3(8.8%)	6(10.7%)

The upstage rate of invasive carcinoma in trial eligible patients would have been 12.9% (21/163) for COMET, 8.8% (3/34) for LORIS, and 10.7% (6/56) for LORD cases, respectively. This rate was lower than the upstaging rate in the general study population (14.3%). Interestingly, all the upstaged cases were ER-positive and HER2 negative.

### Ipsilateral breast cancer events (IBE) at 5 years of follow-up in trial eligible patients

The recurrence rates in the trial eligible patients (in spite of the standard of care therapy) at 5 years follow-up was 11.5% (13/113) for the COMET Trial, 11.1% (3/27) for the LORIS Trial, and 11.9% (5/42) for the LORD Trial (Table 4). The incidence of ipsilateral invasive carcinoma was similar in the entire population and COMET eligible patients (6.6 and 6.2% respectively) but lower in LORIS and LORD trial eligible patients (3.7 and 4.8% respectively).

**Table 4:** Incidence of ipsilateral breast cancer events at least 5 years of follow-up in entire cohort and subset of trial eligible patients.

	Entire population (*n* = 243) No. (%)	COMET eligible (*n* = 113) No. (%)	LORIS eligible (*n* = 27) No. (%)	LORD (new) eligible (*n* = 42) No. (%)
Total	27 (11.1%)	14 (12.4%)	3 (11.1%)	5 (11.9%)
Invasive ductal carcinoma	16 (6.6%)	7 (6.2%)	1 (3.7%)	2 (4.8%)
DCIS	10 (4.1%)	7 (6.2%)	2 (7.4%)	3 (7.1%)
LCIS	1 (0.41%)	0	0	0

## Discussion

The presence of invasive carcinoma is a major concern after a biopsy diagnosis of DCIS. Many studies have analyzed the upstaging rates in this situation and found them to be in the range of 8 to 42.7%^5,10–13^. The higher incidence in some studies may be attributed use of screening methods and type of biopsy resulting in limited tissue sampling and under-diagnosis of invasive disease. Multivariate analysis in our study revealed that the presence of imaging noticed/ palpable mass lesion is an independent predictor of invasive carcinoma in patients with an initial diagnosis of DCIS, which is in agreement with the published literatures^7,23–26^. However, additional radiomics analysis of the images in patients without mass provides only a minor degree of efficacy (“greater than chance”) in identifying invasive disease^27^. Consistent with prior literature^5,7,13^, in our study patients with multiple lesions and histologic high-grade DCIS had a strong trend for upstaging at excision. The presence of comedo necrosis has been associated with upstaging in some studies^11,28^. However, akin to the current study, a meta-analysis involving 7350 cases diagnosed with DCIS^29^ did not find an association between comedo necrosis with upstaging. The reasons for these discrepant results could be the subjectivity of pathologists in making the diagnosis of comedo necrosis^30^ and the strong association of comedo necrosis with grade.

The upstaging rates for the COMET, LORIS, and LORD eligible patients were 12.9, 8.8, and 10.7% respectively; this was slightly lower than the 14.5% in the entire cohort. The overall upstaging rates are at the lower end of the broad range reported in literature^5,10–13^. Grimm et al.^5^ observed a lower upstaging rate of 7% for their COMET eligible patients. The lower incidence in their study could be related to the overall frequency of high-grade DCIS on biopsy (64 versus 41.3% in the current study). Furthermore, both these studies excluded patients with comedo necrosis. In our cohort, it would not have significantly changed the upstaging rate (12.3 vs. 12.9%). For the LORIS eligible patients, Pilewskie et al.^31^ reported upstaging rate of 20%, while Grimm et al.^5^ and Mannu et al.^11^ reported upstaging rates of 7 and 10.3% respectively. The differences could be in part to differences in patient demographics and biopsy techniques. Grimm et al.^5^ had restricted their analysis to nine-gauge vacuum-assisted biopsies. The upgrade rates for the LORD eligible patients reported by Grimm et al and Podoll et al.^5,32^ are similar to those observed in the current study.

Our study is unique in its assessment of the incidence of development of IBE at 5 years for COMET, LORD, and LORIS eligible patients. The incidence of development of IBE at five years of follow-up is 11.1% (27/243) for an unselected population which was similar to COMET, LORIS, and LORD trial eligible patients (11.5, 11.1, and 11.9% respectively). Furthermore, these rates were observed even when patients had been “optimally” treated with surgery, radiation, and or endocrine therapy. It is possible that the rates would have been higher for patients who did not receive these interventions.

The expression of HER2 in DCIS has been associated with the presence of co-existing invasive cancer or the “early” development of invasive carcinoma^33–40^. It has been further suggested that HER2-directed therapy may boost the efficacy of radiotherapy (RT)^41^. This has been the basis of the NSABP B-43 clinical trial which randomized patients to RT or RT plus Trastuzumab (RT plus T). The study enrolled 2014 patients of which 114 patients developed ipsilateral (34 invasive carcinomas). The study did not meet its endpoint of 163 events; however, analysis at 5 years did not show improved efficacy of RT plus T (*p* = 0.11). Our findings of all of the cases upstaged to invasive carcinoma were ER+/HER2 negative could provide one explanation for the negative results of the clinical trial.

The limitations of the current retrospective study include patients being biopsied using different biopsy techniques. These could have impacted the observed results. However, a prospective controlled study would be unlikely to provide results prior to the reporting of the ongoing clinical trials. In this vein, it must be noted that LORD and LORIS trials have been converted to registration trials due to poor accrual. The findings underscore the concerns highlighted by Morrow and Winer (18) and identify increased IBE risk as an additional important factor that needs to be taken into consideration. Overall, our findings support the contention that the risk of missing invasive carcinoma by forgoing surgical excision is relatively small (~10%). However, there is a further risk of ~10% of developing invasive carcinoma in the next 5 years. It is currently unclear what levels of risks can be justifiable/sufficient for patients to accept de-escalation therapies. It is necessary for late risks to be considered and presented to patients (and advocacy groups) in addition to physicians who are making these decisions.

In conclusion, patients with DCIS who meet eligibility criteria for the COMET, LORIS and LORD had a slightly lower rate of upstaging to invasive carcinoma at excision. However, this did not translate into a decreased incidence of IBEs after 5 years of follow-up, even after receiving standard of care therapies. This highlights the need for long-term follow-up in the assessment of the risk of IBE and better criteria for identification of the risk for development of IBEs.

## Methods

### Ethics

The study did not contact patients or use biological materials and all relevant ethical regulatory guidelines were followed. Ethical approval including the need for informed consent was explicitly waived by Indian University Institutional Review Board.

### Cohorts

After obtaining a waiver of consent from Institutional Review Board, the patients’ medical records from Indiana University Medical Center from January 1, 2010 to December 31, 2014 were retrospectively reviewed. The needle biopsies, mostly vacuum-assisted biopsies, had been performed by stereostatic and/or ultrasound-guided methods. Several potential variables including age at diagnosis, race, family history of breast cancer, indication for mammography, breast symptoms (nipple discharge and presence of mass lesion), surgical excision, and post-surgical treatments were collected. Patients diagnosed with DCIS with prior or synchronous invasive ductal carcinoma including DCIS with micro-invasion and those who did not undergo surgical excision were excluded. Individual demographic and clinical parameters of DCIS cases were listed in Supplementary Table 1.

Surgical pathology reports from the initial core needle biopsy and subsequent surgical excision were reviewed for upstaging to invasive disease and disease recurrence. All cases are signed out by a small group of pathologists at Indiana University. Tumor histological grade, histological architecture (cribriform, papillary, micropapillary, and solid), presence of microcalcification and necrosis including comedo necrosis, the status of estrogen receptor (ER), and progesterone receptor (PR) were recorded. For cases with missing receptor status on initial needle biopsy, the results from the surgical excision were used. Mammography reports were reviewed to identify the findings leading to the needle biopsy (such as microcalcification or associated mass). Lesion size, location, and number of cores at biopsy were not analyzed in the present study because of inconsistent and insufficient reporting of these variables. All patients had complete excision with negative margins as per medical records. It is our institutional practice to obtain 3 mm margins for DCIS, with exceptions being made for specific conditions such as (deep) fascial and (superficial) skin margins.

Cases were classified by eligibility criteria for the COMET, LORIS, and LORD DCIS active surveillance trials as summarized in Supplementary Table 2. The rate of upstaging to invasive carcinoma and rate of developing new breast neoplastic lesions during follow-up were calculated for cases that met published eligibility criteria for each trial^20–22^.

### Statistical methods

Descriptive statistics were used to present the characteristics of the population. Due to the nature of binary categorical response factors, logistic modeling was performed to regress demographic and pathology variables on invasive disease upstaging and recurrence rates, respectively. Univariate analysis was performed to regress individual variables, followed by multivariable analysis. The numeric variables such as, age, were converted to a categorical variable by classifying ages into two groups (<45 and ≥45). Separate logistic regression models were constructed for patients upstaging to invasive carcinoma vs. recurrent with new breast lesions. Odds ratios, defined as *p*/(1-*p*), where *p* is the probability of a response occurring, were computed for each variable. For any logistic regression model, the odds ratio for the *i*th predictor variable is the exponential of the *i*th coefficient. A 95% confidence interval was estimated for each predictor coefficient. *p* value of 0.05 was considered statistically significant. All analyses were performed using the MATLAB R2019b (MathWorks, MA, USA).

### Reporting Summary

Further information on research design is available in the Nature Research Reporting Summary linked to this article.

## Supplementary information

Reporting Summary Checklist

Suppl Table 1

Suppl Table 2

## Data Availability

The data that support the findings of this study are available from the corresponding author upon reasonable request.
